# “WE ARE SYMBIOTIC”: THE IMPACT OF MULTIPLE CHRONIC CONDITIONS ON RELATIONSHIP DYNAMICS AMONG OLDER COUPLES

**DOI:** 10.1093/geroni/igae098.3003

**Published:** 2024-12-31

**Authors:** Angelica Eagle, Courtney Polenick

**Affiliations:** University of Michigan, Ann Arbor, Michigan, United States; University of Michigan, Ann Arbor, Michigan, United States

## Abstract

Despite the growing recognition of the impact of multiple chronic conditions (MCC) on individuals’ lives and relationships, there is limited understanding about the effects on spousal relationships when both spouses live with MCC. This qualitative study evaluated how older couples living with MCC perceive the impact of their conditions on their relationship. A total of 56 wives (M = 66.84 years, SD = 9.73) and husbands (M = 68.16 years, SD = 10.69), who both reported having two or more chronic conditions, completed separate semi-structured interviews that included open-ended questions about the effect of their conditions on their relationship. We separately evaluated couples that included a partner living with early-stage dementia (n = 15) and couples not living with dementia (n = 41) to assess whether the presence of dementia affects couples with MCC differently due to distinct care needs and communication challenges. The data were coded using qualitative content analysis to develop major themes. Themes for the relationship impacts of MCC among couples not living with dementia included: 1) adapting daily routines around functional limitations; 2) changes in emotional and physical intimacy; 3) changes in shared activities; and 4) stronger relationship impact on one partner. Themes for couples living with dementia included: 1) changes in emotional and physical intimacy; 2) stronger relationship impact on spouses of people living with dementia; and 3) increased time spent together. These findings identify potential targets for dyadic interventions to improve relationship functioning and quality among older couples living with MCC.

